# Complex I deficiency and Leigh syndrome through the eyes of a clinician

**DOI:** 10.15252/emmm.202013187

**Published:** 2020-10-30

**Authors:** Karit Reinson, Katrin Õunap

**Affiliations:** ^1^ Department of Clinical Genetics Institute of Clinical Medicine University of Tartu Tartu Estonia; ^2^ Department of Clinical Genetics United Laboratories Tartu University Hospital Tartu Estonia

## Abstract

Mitochondrial complex I deficiency is associated with a wide range of clinical presentations, including Leigh syndrome. Its genetic causes are heterogeneous, with poor genotype–phenotype correlation. It is impossible to identify the genetic defect of complex I deficiency using clinical observation and metabolic/imaging studies alone. As a result, whole‐exome sequencing (WES) is increasingly used in clinical work to identify an underlying genetic defect causing the disease. The article in this issue of *EMBO Molecular Medicine* by Alahmad *et al* (2020) is timely and valuable, as it expands on the genotype of mitochondrial complex I deficiency by identifying and characterising pathogenic variants of the *NDUFC2* gene in children with Leigh syndrome.

## Graphical abstract

K. Õunap and K. Reinson discuss the biochemical and functional characterization of the *NDUFC2* pathogenic variants identified in children with Leigh syndrome by R. Taylor and colleagues, in this issue of *EMBO Molecular Medicine*

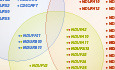

Mitochondria are present in all nucleated cells and responsible for the generation of adenosine triphosphate (ATP) via oxidative phosphorylation (OXPHOS). Around 90% of the cell energy requirements are achieved through hydrolysis of ATP, making the mitochondrial ATP production a crucial energy source for the human body (Harris & Das, 1991). The OXPHOS system contains five multi‐subunit complexes and two electron carriers. In mammals, mitochondrial complex I is composed of 45 subunits and is responsible for the most frequently observed single‐enzyme deficiency causing OXPHOS disorders (Rodenburg, 2016). Clinical phenotypes associated with complex I deficiencies include Leigh syndrome, severe or fatal lactic acidosis, leukoencephalopathy, pure myopathy, hepatopathy with renal tubulopathy, neonatal cardiomyopathy, Leber’s hereditary optic neuropathy and mitochondrial encephalomyopathy with lactic acidosis and stroke‐like episodes (Fassone & Rahman, 2012). However, there is increasing evidence that the phenotype of complex I deficiency is even broader including, for example, isolated congenital sideroblastic anaemia (Lichtenstein *et al*, 2016). Early symptoms often present a non‐specific clinical and biochemical picture, regardless of patient age, and phenotypes may vary widely among family members (Reinson *et al*, 2019).

Leigh syndrome, first described by Denis Leigh in 1951 as a subacute necrotising encephalomyelopathy, is a rare inherited progressive neurodegenerative disorder first. It is characterised by focal, bilaterally symmetrical and subacute necrotic lesions in the thalamus, brainstem and posterior columns of the spinal cord. As there is no single clinical or laboratory criterion, diagnosis of Leigh syndrome is based on clinical observation, family history, laboratory evaluations, imaging, histochemical staining of muscle biopsies, mitochondrial respiratory chain enzyme activity analysis and identification of mitochondrial DNA (mtDNA) or nuclear DNA (nDNA) pathogenic variant(s) (Baertling *et al*, 2014). The most frequently observed abnormality, which occurs in more than 30% of patients, is complex I deficiency (Fassone & Rahman, 2012). Disease onset is typically between 3 and 12 months with 50% of affected individuals dying at 3 years of age (Rahman *et al*, 1996). However, Leigh syndrome can also occur during adolescence or adulthood (Baertling *et al*, 2014), and with appropriate treatment, patients can survive for many years after diagnosis (Rahman *et al*, 1996).

Mitochondrial disorders are caused by pathogenic variant(s) in either nDNA or mtDNA. To date, there are 89 genes known to cause Leigh syndrome (Rahman *et al*, 2017) and at least 44 genes encode complex I subunits. Figure 1 shows the correlation between nuclear‐encoded complex I subunit and assembly factor genes and the main clinical phenotype (neurological, metabolic, cardiac and exercise intolerance). Identification of novel homozygous *NDUFC2* variants in 3 subjects with Leigh syndrome by Alahmad *et al* adds *NDUFC2* to the list of genes causing complex I deficiency (Fig 1). Furthermore, Alahmad and colleagues revealed that the mutations in *NDUFC2* gene cause a defect in the assembly of the complex I holoenzyme suggesting an important role for *NDUFC2* in the assembly of the membrane arm of complex I (Alahmad *et al*, 2020).

**Figure 1:**
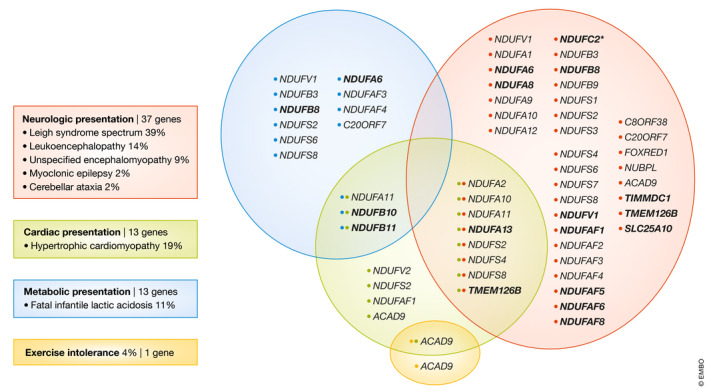
The correlation between nuclear‐encoded genes and the clinical phenotype The correlation between nuclear‐encoded complex I subunit and assembly factor genes and the main clinical phenotype (neurological, metabolic, cardiac and exercise intolerance) is given in this Venn diagram. This diagram, and the relative prevalence of the main clinical phenotypes, is adapted from the Fassone and Rahman review. The 16 new genes, identified after 2012, are marked in bold. Asterisk (^*^) signifies a gene identified by Alahmad *et al*(2020).

From a clinician's point of view, treatment especially of a multi‐systemically affected child with mitochondrial disease is complex and must be rapid. It is important to highlight that the precise molecular diagnosis allows clinicians to counsel the patients and their families about the possibilities of treatment, recurrence risk, prenatal testing options and prognosis. The introduction of WES in clinical work has greatly improved the prospect of achieving a genetic diagnosis for patients with high clinical diagnostic scoring of mitochondrial disorders in up to 60% of cases (Puusepp *et al*, 2018). However, WES does not magically produce an effortless diagnosis in all cases. Data may not provide enough certainty for a definitive diagnosis, and a clinician may have few opportunities (and little time) for fundamental and broad‐based functional research. In complicated and fast‐evolving cases, bedside decisions on patient care still rely on the literature.

The value and importance of the article by Alahmad *et al* lie in functional studies with new pathogenic variants of the *NDUFC2* gene. From a physician’s point of view, identification of these gene variants will support prenatal diagnosis and allow a more accurate prognosis that would offer appropriate family counselling that is often the only relief in a difficult time. In addition, Alahmad *et al* also impart detailed information on gene‐encoded proteins, mutation‐induced dysfunctions and complex I assembly pathways, providing a basis for future research and hopefully treatment options as well.

In conclusion, we still do not completely understand the complexity of diseases and their causes. Therefore, collaboration between clinicians and scientists in the areas of genomics, transcriptomics, proteomics and metabolomics is essential for the continued development of evidence‐based diagnoses and treatments.

## References

[emmm202013187-bib-0001] Alahmad A , Nasca A , Heidler J , Thompson K , Oláhová M , Legati A , Lamantea E , Meisterknecht J , He L *et al* (2020) Bi‐allelic pathogenic variants in *NDUFC2* cause early ‐onset Leigh syndrome and stalled biogenesis of complex I. EMBO Mol Med 12: e12619 10.15252/emmm.202012619 PMC7645371 32969598

[emmm202013187-bib-0002] Baertling F , Rodenburg RJ , Schaper J , Smeitink JA , Koopman WJ , Mayatepek E , Morava E , Distelmaier F (2014) A guide to diagnosis and treatment of Leigh syndrome. J Neurol Neurosurg Psychiatr 85: 257–265 10.1136/jnnp-2012-304426 23772060

[emmm202013187-bib-0003] Fassone E , Rahman S (2012) Complex I deficiency: clinical features, biochemistry and molecular genetics. J Med Genet 49: 578–590 22972949 10.1136/jmedgenet-2012-101159

[emmm202013187-bib-0004] Harris DA , Das AM (1991) Control of mitochondrial ATP synthesis in the heart. Biochem J 280(Pt 3): 561–573 1837214 10.1042/bj2800561 PMC1130493

[emmm202013187-bib-0005] Lichtenstein DA , Crispin AW , Sendamarai AK , Campagna DR , Schmitz‐Abe K , Sousa CM , Kafina MD , Schmidt PJ , Niemeyer CM , Porter J *et al* (2016) A recurring mutation in the respiratory complex 1 protein NDUFB11 is responsible for a novel form of X‐linked sideroblastic anemia. Blood 128: 1913–1917 27488349 10.1182/blood-2016-05-719062 PMC5064715

[emmm202013187-bib-0006] Puusepp S , Reinson K , Pajusalu S , Murumets U , Oiglane‐Shlik E , Rein R , Talvik I , Rodenburg RJ , Ounap K (2018) Effectiveness of whole exome sequencing in unsolved patients with a clinical suspicion of a mitochondrial disorder in Estonia. Mol Genet Metab Rep 15: 80–89 30009132 10.1016/j.ymgmr.2018.03.004 PMC6043467

[emmm202013187-bib-0007] Rahman S , Blok RB , Dahl HH , Danks DM , Kirby DM , Chow CW , Christodoulou J , Thorburn DR (1996) Leigh syndrome: clinical features and biochemical and DNA abnormalities. Ann Neurol 39: 343–351 8602753 10.1002/ana.410390311

[emmm202013187-bib-0008] Rahman J , Noronha A , Thiele I , Rahman S (2017) Leigh map: a novel computational diagnostic resource for mitochondrial disease. Ann Neurol 81: 9–16 27977873 10.1002/ana.24835 PMC5347854

[emmm202013187-bib-0009] Reinson K , Kovacs‐Nagy R , Oiglane‐Shlik E , Pajusalu S , Noukas M , Wintjes LT , van den Brandt FCA , Brink M , Acker T , Ahting U *et al* (2019) Diverse phenotype in patients with complex I deficiency due to mutations in NDUFB11. Eur J Med Genet 62: 103572 30423443 10.1016/j.ejmg.2018.11.006

[emmm202013187-bib-0010] Rodenburg RJ (2016) Mitochondrial complex I‐linked disease. Biochim et Biophys Acta 1857: 938–945 10.1016/j.bbabio.2016.02.012 26906428

